# Cognition Impairment and Gait Disorders in Older Adults

**DOI:** 10.3390/ijerph19127347

**Published:** 2022-06-15

**Authors:** Patrick Manckoundia, France Mourey

**Affiliations:** 1 Department of Geriatrics and Internal Medicine, Hospital of Champmaillot, University Hospital, 21000 Dijon, France; 2 UMR Inserm/U1093 Cognition, Action, Sensorimotor Plasticity, University of Burgundy and Franche Comté, 21000 Dijon, France; france.mourey@u-bourgogne.fr

**Keywords:** Alzheimer disease, balance, falls, gait, major neurocognitive disorder, older adult

Thanks to the increase in life expectancy linked to scientific and medical progress and improvements in hygiene conditions, the population of people aged 75 years and over continues to grow worldwide, particularly in industrialized countries. These older individuals can age either normally or pathologically. Individuals with pathological ageing have numerous comorbidities, leading to polypharmacy and contributing to frailty. Among these comorbidities, major neurocognitive disorders (MNCDs) and balance and gait disorders are two of the main public health problems. Alzheimer’s disease (AD) is the most common MNCD [1]. Its prevalence is 44 million cases worldwide, a number which is expected to double by 2050 [2]. In addition to AD, the other most common MNCDs in the older population are vascular MNCD, Lewy body disease, MNCD secondary to Parkinson’s disease and frontotemporal lobar degeneration [3]. In older adults, MNCDs are the primary cause of loss of autonomy, i.e., the ability to govern oneself, while balance and gait disorders are a major factor in loss of independence, and falls are the most common cause of accidental death. Moreover, cognitive troubles in older adults with MNCDs, especially AD, are often associated with motor disorders, including bradykinesia, rigidity, and balance and gait disorders [4,5], which increases the risk of falls [1]. The increased risk is explained by the impaired judgment (including the ability to assess environmental risks), memory and/or praxis and/or attention disorders (especially in a multitasking situation with poor integration of simultaneous stimuli), and damage to the nerve centers governing balance and gait that occur in individuals with MNCDs [6,7]. There is also the negative impact of sarcopenia on motor skills, especially in older adults with MNCD. Indeed, a close link has been confirmed between sarcopenia and MNCDs, in particular AD [5]. Several previous studies have shown associations between slowing gait speed and cognition, suggesting that slow gait speed precedes cognitive decline by several years in people who will develop MNCD [5]. The motoric cognitive risk syndrome, which associates slow gait speed and subjective memory complaints without objective cognitive and functional disorders, illustrates this association [8].

In view of the observations described herein, we propose this Special Issue to provide experts in MNCDs and/or balance and gait disorders in older adults the possibility to publish original or review articles. The objective is to enhance our understanding of the relationship between these two pathological situations, with the ultimate goal of improving care and quality of life in the affected population.
